# Application of Stem Cells in Orthopedics

**DOI:** 10.1155/2012/394962

**Published:** 2012-02-23

**Authors:** Andreas Schmitt, Martijn van Griensven, Andreas B. Imhoff, Stefan Buchmann

**Affiliations:** ^1^Department of Sports Orthopedics, Technical University, 81675 Munich, Germany; ^2^Department of Trauma Surgery, Technical University, 81675 Munich, Germany; ^3^Department of Orthopedic Surgery, Schulthess Clinic, 8008 Zurich, Switzerland

## Abstract

Stem cell research plays an important role in orthopedic regenerative medicine today. Current literature provides us with promising results from animal research in the fields of bone, tendon, and cartilage repair. While early clinical results are already published for bone and cartilage repair, the data about tendon repair is limited to animal studies. The success of these techniques remains inconsistent in all three mentioned areas. This may be due to different application techniques varying from simple mesenchymal stem cell injection up to complex tissue engineering. However, the ideal carrier for the stem cells still remains controversial. This paper aims to provide a better understanding of current basic research and clinical data concerning stem cell research in bone, tendon, and cartilage repair. Furthermore, a focus is set on different stem cell application techniques in tendon reconstruction, cartilage repair, and filling of bone defects.

## 1. Introduction

Today great hope is set on regenerative medicine in all medical fields. Leland Kaiser introduced the term “Regenerative medicine” in 1992. He forecasted that “a new branch of medicine will develop that attempts to change the course of chronic diseases and in many instances will regenerate tired and failing organ systems” [1]. Since then, scientists all over the world try to develop cell-based approaches to regenerate damaged tissues, or even substitute whole organs [2].

Of course, regenerative medicine has developed to be of interest in orthopedics. There, great hope was set on regenerative medicine to develop alternative therapies for cartilage damage, arthritis, large bone defects, or atrophic tendon ruptures during the last decade. These are all indications, which are treatable only insufficiently with conventional implants and surgical procedures [3–10]. Therefore, they frequently result in decreased function of the musculoskeletal system or even loss of patients' mobility. In the worst case, the mentioned diseases even result in a loss of autonomy for the patient. In consequence, this implies immense costs for the health care systems all over the world.

In this review, we focus on application of stem cells in regenerative medicine for orthopedic indications. We present current approaches in stem cell-based therapy in orthopedics and review recent successes in basic science and clinical application of regenerative medicine approaches within the field.

## 2. Stem Cells

Stem cells are of particular interest in regenerative medicine. They inhere several unique characteristics that distinguish them from other cell types. Stem cells represent unspecialized cells, which have the ability to differentiate into different adult cell types. Here, it is important to distinguish embryonic stem cells, which are truly pluripotent from multipotent adult stem cells. Embryonic stem cells (ESCs) are only found in early developmental stages of the organism. They represent the only cell type, which has the ability to renew itself indefinitely and is truly pluripotent. As a unique precursor cell, it can differentiate into cells of all three germ layers [2]. In contrast, a variety of multipotent adult stem cells exists in assumedly all tissues of the organism. They are responsible for maintaining the integrity of the tissue they reside in. Usually, these adult stem cells show limited differentiation potential to tissues of one germ layer [2].

The use of human ESCs as a resource for cell therapeutic approaches is currently an intensively researched field [11–13]. From a legal and ethical point of view, research involving human embryonic cells is highly controversial and many countries are reviewing their legislation. Besides the ethical concerns, the use of embryonic stem cells is problematic, as the application of allogenic pluripotent cells inheres a distinct oncogenic potential that currently forbids the application in patients.

The work of Takahashi and Yamanaka in 2006 has opened new perspectives in regenerative medicine. His group was the first to demonstrate successful dedifferentiation of somatic cells into a pluripotent ESC-like status by transfection with four embryonic transcription factors [14]. The so-called induced pluripotent stem cells (iPS cells) provide the possibility of autologous therapy with pluripotent and easily accessible cells in the future. Beside the great potential this technique undoubtedly represents, it bears some essential safety problems which are currently far from being solved. As ESCs, these cells inhere a high oncogenic potential which currently forbids application in patients. If they are injected in an undifferentiated state, they cause teratomas, and mice generated from iPS cells show high rates of tumors. This oncogenicity may be due to the transcription factors used for dedifferentiation which are known to be oncogenes, due to the insufficient epigenetic remodeling or due to the oncogenic retroviruses used for transfection [15].

The use of adult stem cells raises less ethical concerns and has proved to be much safer than pluripotent stem cells. In addition, these cells have further advantages compared to ESCs, for example, a use for autologous cell therapies, using patients' own cells to reduce possible immune responses, is easier to realize. Nonetheless, the limited differentiation potential of adult stem cells narrows their applicability. Typically, adult stem cells can differentiate into the cell types of the tissue in which they reside. Mesenchymal stem cells have been found to be the most promising candidates, as they show good differentiation potential towards cartilage, tendon and bone cells. They can be isolated from a number of mesenchymal tissues as for example bone marrow, fat, synovial membrane, periosteum, and others [16]. Interestingly, these mesenchymal stem cells have been found to differ regarding their differentiation potential dependent on their tissue source [17].

As ethical and safety concerns currently forbid application of iPS cells and ESCs in patients [2, 18], we will focus on adult mesenchymal stem cells within the rest of the paper.

## 3. Application of Mesenchymal Stem Cells in Regenerative Medicine

**Figure 1:**
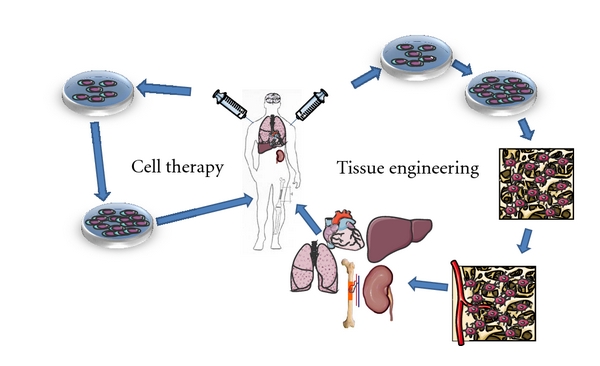
The two strategies of stem cell application in regenerative medicine. Stem cells are either isolated from the patient (autologous transplantation) or from other donors (allogenous transplantation). The cells are expanded *in vitro* and either applied directly to the patient to substitute lost cells (“cell therapy”), or seeded into 3 dimensional scaffolds (“Tissue engineering”) and differentiated into the demanded cell type. The composed artificial tissue construct is subsequently implanted into patients' tissue defect.

Regenerative medicine mainly includes two different strategies of cell-based therapy. In the first approach, cells are applied to substitute damaged cells within a tissue to reconstitute its integrity and function. During this procedure called “cell therapy” a cell suspension is simply injected into the damaged tissue or into the blood circulation. The second approach called “tissue engineering” is more complex. Here, cells are combined with a three dimensional matrix to compose a tissue-like construct to substitute lost parts of the tissue, or even whole organs (Figure 1) [2].

One of the most successful examples in “cell therapy” is the transplantation of hematopoietic stem cells. This procedure has now been practiced for decades to treat serious hematological diseases. For transplantation of bone marrow, hematopoietic stem cells are injected into the blood circulation of the recipient. Interestingly, they find their way to the bone marrow by a phenomenon termed “homing.” Chemokines were found to play a key role in homing of hematopoietic stem cells [19–21].

Several experiments have proven the ability of homing to injured tissue for several types of stem cells. In animal models of hepatic intoxication, partial hepatectomy, myocardial infarction, nephropathy, cerebral ischemia, lung injury, lung fibrosis, and local irradiation, stem cells enriched in injured tissue and partially differentiated into tissue-specific cell types after systemic injection [22–38]. Cell therapy with systemically injected mesenchymal stem cells was also performed in humans, showing beneficial effects in graft-versus-host disease or osteogenesis imperfecta [39, 40].

However, cell therapy alone is not sufficient to regenerate large tissue defects or even replace whole organs. Therefore, the approach of “tissue engineering” is the more promising strategy. In the process, tissue-specific cells are seeded on a scaffold imitating the architecture of the tissue-specific extracellular matrix. In the last decade, basic science has made great advantages in tissue engineering research, resulting in *in vitro* composition of multiple different functional tissue constructs [41]. Nonetheless, tissue engineering therapy has barely reached the patient [42]. The reason for the modest entering of tissue engineering methods into the clinic is the yet unsolved problem of vascularization [43]. Thus, an intact vascular network is a prerequisite to realize tissue constructs of more than 400 *μ*m in diameter [44]. In the last decade many scientists in the field of tissue engineering have focused on solving the problem of vascularization. However, all efforts proving applicability for tissue engineering of large solid tissues or even whole organs in humans have failed so far (for paper see [43]). Nonetheless, tissue engineering was already successfully used in patients to substitute either hollow organs with limited wall diameter (trachea, bladder) or avascular tissues as cartilage [45–47]. In these cases, the diffusion trajectory is sufficient to maintain cell survival.

## 4. Participation of Mesenchymal Stem Cells in Tissue Regeneration

**Figure 2:**
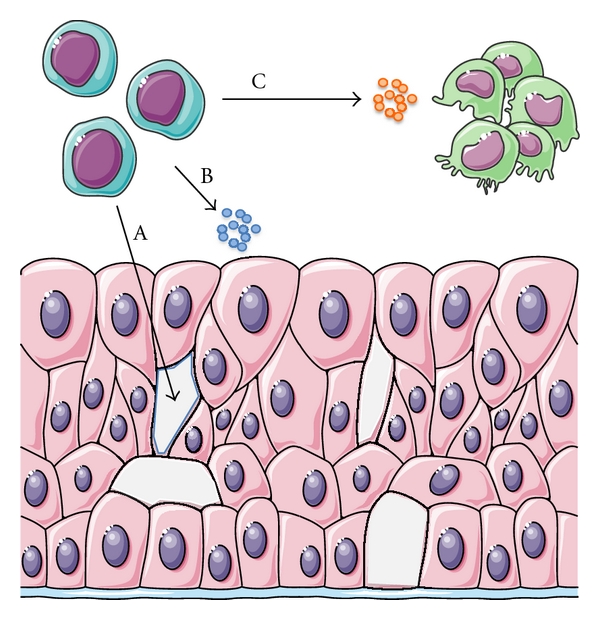
Stem cells participate in tissue regeneration in different ways. They directly differentiate into tissue-specific cells and thus substitute damaged or lost cells (A). They indirectly influence tissue regeneration by secretion of soluble factors. Here they promote vascularization, cell proliferation, differentiation within the tissue (B) and modulate inflammatory processes (C).

Mesenchymal stem cells have the ability to migrate chemotactically to tissues showing inflammation and injury in the organism [48]. Besides their unique ability to differentiate into different cell types, mesenchymal stem cells were found to secrete a variety of cytokines, showing anti-inflammatory activity and create an anabolic microenvironment [17]. Furthermore, direct cell-cell contact immunomodulation has also been shown. Thus, they participate in regeneration of injured tissues in different ways. On one hand, they directly differentiate into tissue-specific cells and thus substitute damaged or lost cells. On the other hand, they indirectly influence tissue regeneration by secretion of soluble factors. Thirdly, they are able to modulate the inflammatory response. Thus, they can promote vascularization, cell proliferation, differentiation and modulate an inflammatory process (Figure 2).

Indeed, there is evidence for all mentioned activities of MSCs in tissue regeneration from *in vitro* and *in vivo* experiments. The differentiation potential of MSCs was extensively studied *in vitro*. The cells were found to inhere the potential of multilineage differentiation towards possibly all kinds of mesenchymal cells such as cartilage, bone, tendon, and fat cells, and fibroblasts [69]. Excitingly, further studies revealed that differentiation capacity of MSCs seems not to be restricted to cells belonging to the mesenchymal lineage. They were shown to be able to differentiate towards cells from other germinal layers, as for example, neurons, glia cells, cardiomyocytes, endothelial cells and hepatocytes [70–73]. *In vivo* experiments and first clinical applications confirmed the ability of MSCs to engraft within a variety of injured tissues and differentiate into tissue-specific cells and thus substitute lost cellular function [17, 74].

In many studies, beneficial effects appeared without any detectable engraftment of the applied mesenchymal stem cells to the damaged tissue, however. Moreover, MSC protein extracts and conditioned medium from MSC cultures showed similar improvement of organ function in liver disorders or heart ischemia [75, 76]. Further investigation of MSCs revealed that they release paracrine factors for example IGF-1, HGF, VEGF, IGF-2, bFGF, or pre-microRNAs which protect's host cells, promote cell proliferation and enhance angiogenesis [77, 78]. These positive effects could partially be confirmed *in vivo*, where MSCs activated expression of some of the mentioned factors in the myocardium and promoted angiogenesis [79]. Furthermore, MSCs secrete paracrine factors which enhance lung function by regulating endothelial and epithelial permeability, decreasing inflammation, enhancing tissue repair, and inhibiting bacterial growth in acute lung injury and acute respiratory distress syndrome [80]. Beneficial effects of paracrine MSC signaling could also be confirmed in healing of cutaneous wounds [81]. The recently identified potential of paracrine MSC signaling on damaged tissue even caused some authors call MSCs an “injury drug store” [82].

Besides their mentioned differentiation potential and their ability to promote tissue regeneration by secretion of soluble factors, MSCs inhere extraordinary immunological properties. There is increasing evidence that the cells themselves are relatively nonimmunogenic and they can be readily transplanted between different individuals without initiating an immune response [83]. Furthermore, they proved to inhere anti-inflammatory and immunosuppressive capability *in vitro* and *in vivo*, where they can modulate immune responses on different targets. They inhibit maturation of immune cells, like helper T, cytotoxic T, dendritic, and B cells. Additionally, cells express a number of cytokines that can suppress inflammation, as for example TGF-beta1, NO, prostaglandin-E2, HLA-G, hepatocyte growth factor, and IL-10 [17]. The revealed anti-inflammatory effects of MSCs have opened a broad field of possible applications in transplantation and immune disorders. After confirming the anti-inflammatory effects in several animal models, first promising clinical applications have succeeded. In these applications, MSCs showed beneficial effects on graft versus host disease after hematopoietic stem cell transplantation and on Crohn's disease [84]. However, first results are promising and the *in vivo* application seems to be rather safe, as no serious side effects have been reported. Nonetheless, randomized trials have to follow to confirm these first results.

## 5. Application Techniques in Orthopedics

To differentiate between favorable application strategies the aim of treatment is one important factor. As mentioned before, MSCs have the potential to rebuild injured tissue but also to secrete growth factors for enhancing tissue regeneration. Depending on the underlying pathology, the treatment strategies differ considerably. In one patient a large tissue defect has to be filled by means of tissue engineering, whereas in another the substantial defect is bridged with residual tissue of low quality and only an improvement of healing environment is indicated.

Besides the direct injection in the surrounding tissue, biomaterials are frequently used as carriers for drugs, bioactive molecules and cells. These materials have to fulfill some fundamental requirements. At first they have to be immune-compatible and nontoxic, whereas the degradation process must neither release toxic substances nor tissue-toxic concentrations of degradation products. For a later replacement with regenerated tissue, bio-degradable materials are important. The degradation velocity must be balanced as too fast and too slow are both detrimental. Beside these qualities, matrices formed from biomaterials must have distinct properties with regard to the desired kind of tissue. The prerequisite of mechanical strength, bioactivity, and kinetics of degradation and drug/cell release significantly varies between different repair tissues. Besides the used biomaterials themselves, the 3-dimensional structures of scaffolds have great influence on cell growth and differentiation. Scaffolds must be highly porous with interconnected pores of a diameter of at least 100 *μ*m to allow ingrowth of cells and vessels [85]. Pore sizes between 100 and 400 *μ*m are ideal.

Despite the tissue engineering of bone, for which various inorganic materials, such as hydroxyapatite, calcium phosphate, calcium carbonate, or glasses was tested, mainly organic biomaterials have been investigated for scaffold formation. These are either naturally derived, for example, collagen, fibrin, agarose, alginate, gelatin, silk or hyaluronic acid, or produced synthetically. Synthetically produced organic biomaterials are mainly polyhydroxyacids such as polyglycolides or polylactides. To control kinetics of degradation, recent studies were performed employing hydroxyl acid copolymers. Thus, it has been tried to adapt kinetics of degradation to those of tissue regeneration.

As these synthetic polymers often lack bioactivity, their surface was modified to alter cell adhesion, migration, differentiation, and proliferation in recent studies. Thus, they were coated or copolymerized with bioactive materials or functional groups were attached to the polymer chain before scaffold fabrication [86–88]. Apart from surface modifications with bioactive materials, scaffolds were coated directly with cytokines to control proliferation and differentiation of seeded cells [89]. Other authors describe the coating of scaffolds with genetic vectors to perform transfection of cells with different growth factors [90]. Biomaterials for tissue engineering can also carry drugs that prevent microbial colonization or control ingrowth of scaffolds into the surrounding tissue [91, 92].

### 5.1. Tendon Repair

Considering physiological properties of tendon tissue, an application technique via scaffolds with native extracellular matrix and the capability of cell seeding and adhesion would be ideal [93]. Based on this hypothesis, most of the current studies used scaffold application techniques. The few studies which favored direct application techniques injected the suspension of MSC into bone tunnels or on the bone surface before tendon refixation to improve tendon-to-bone healing [94, 95].

Scaffold application techniques for tendons can be divided into gel suspensions, 3D scaffolds of solid tissue, and hybrid techniques. Gel suspensions offer a perfect 3D filling of the defect, but the reduced stability in comparison to stable matrices may result in loss of gel at the repair site due to erosion. In a rabbit Achilles tendon model, Chong et al. [96] used a mixture of fibrin sealant and bone marrow-derived mesenchymal stem cells. The fibrin sealant was injected into the tendon and the repair site was additionally covered with the agent. Fibrin incorporates the advantages of a clinical use over years including FDA approval, bone marrow-derived mesenchymal stem cells remain viable in fibrin and published data indicate that fibrin itself has no effect on tendon healing [97]. In this study no differences between fibrin and fibrin with MSC could be shown histologically. In the early healing phase (3 weeks), significantly improved biomechanical properties were documented but not in subsequent time periods (6 and 12 weeks). In a rat rotator cuff model, Gulotta et al. [98] also used MSC in a fibrin sealant and placed it between tendon and bone before refixation of the tendon. In this acute tendon repair model they did not find any significant histological or biomechanical differences after 2 or 4 weeks, respectively. Noteworthy, the same group recently succeeded in enhancing tendon healing in the same rotator cuff model, applying transfected MSCs using the embryonic transcription factor MT1-MMP and the tendon transcription factor scleraxis [99, 100]. With a collagen gel, Awad et al. [101] presented a further gel-based application technique. They fixed a collagen gel with different concentrations of MSC to suture material and filled a defect in the rabbits' patellar tendon. After 12 and 26 weeks, significantly higher maximum stresses and moduli were documented compared to natural repair tissues. However, an adverse event was observed as there had been an increased number of intratendinous ossifications (28%). In comparison to the intact tendon only 25% of the ultimate load was reached with MSC. Regarding all groups, cell concentration had no significant influence on the outcome. This study group improved its application technique and presented a hybrid technique (MSC in a gel-collagen sponge composite) [102]. In the rabbit patellar tendon model, the biomechanical properties and cellular alignment were significantly improved in the MSC group after 12 weeks. A different matrix is presented by Omae et al. in *in vitro* and *in vivo* studies [103, 104]. Xenotendon slices with a thickness of 50 *μ*m were decellularized and seeded with bone marrow stromal cells. The first results of the bundled construct in a patellar tendon rat model showed a survival of the stromal cells in all layers. *In vivo* results with MSC have not been published yet but the approach is promising.

In conclusion, the application of MSC in tendon repair shows promising but inhomogenous results in animal models. Current *in vivo* data favor the culture of MSC into a tissue-engineered construct, with the advantage of primary stability and allowing the cells to produce their own extracellular matrix. But there is no consensus about the ideal carrier construct. Clinical data are not yet available for MSC application in tendon repair.

### 5.2. Cartilage

Besides autograft transplantation and autologous chondrocyte transplantation, current therapeutic concepts of cartilage defects include the recruitment of MSC. Drilling, abrasion, or microfracturing of the subchondral bone aims at the recruitment of MSC from the subchondral bone to stimulate the formation of cartilage repair tissue. In experimental and clinical studies of these standard techniques, a nonhyaline cartilage with high proportions of fibrous elements and inferior functionality has been found [105].

For autologous cartilage repair various two- and three-dimensional constructs are available. Most of the matrices consist of natural polysaccharides and proteins, such as alginate and collagen. Furthermore, synthetic polymers are also available for example, polyethylene glycol (PEG) or polylactic acid (PLA). Successful outcome of a stem cell-based cartilage tissue engineering also depends on the design of extracellular matrix for a proper differentiation of MSCs into chondrocytes [106]. The most important property, namely, mechanical stability, to provide appropriate cell-matrix interactions to stimulate tissue growth and capability of functional tissue growth. The ideal matrix has sufficient strength to protect the cells from axial load and shear forces, is highly adhesive to remain stable in the repair site and possesses enough porosity to allow nutrient and differentiation factors to diffuse through it. Currently, a large number of *in vitro* studies focus on the optimal three-dimensional matrix.

Increasingly innovative matrices are tested in *in vivo* animal models. For example, Shafiee et al. [107] performed MSC-based cartilage repair in a rabbit model with full-thickness cartilage defects. They used poly(vinyl alcohol)/polycaprolactone (PVA/PCL) nanofibers as matrix which showed a support of MSC proliferation and chondrogenic differentiation *in vitro*. The animals treated with MSC showed an improved healing of the defects compared with the untreated control. Tay et al. [108] used alginate-embedded MSC for the repair of focal cartilage defects in a rabbit model. They compared the macroscopic and histological results of MSC versus autologous chondrocyte transplantation 6 months postoperatively. MSCs had a similar effectiveness as chondrocyte transplantation, MSC even showed a significantly better macroscopic score. Both treatments resulted in superior tissue regeneration compared with untreated control defects. These promising results from the laboratory resulted in the first clinical studies about cartilage repair with support of MSC. The earliest data are case series of Wakitani et al. [109, 110]. They performed a bone marrow aspiration from the iliac crest and the MSC were expanded in culture. Four weeks later, the MSC were implanted using a collagen gel and the defect was additionally covered with a periosteal flap. The authors describe satisfying clinical and macroscopic results, but the small number of patients, the retrospective study design and the missing control has to be taken into consideration. Nejadnik et al. [111] performed a matched pair analysis of 36 patients in each group who underwent autologous cartilage transplantation or implantation of MSC. The postoperative followup after 24 months showed no significant difference of different functional knee scores between the groups.

In the treatment of osteochondral lesions, the group of Buda et al. [66, 112] published clinical results of lesions in the femur condyle and the talus. In the talus group, MSC were taken from the iliac crest and incubated with a hyaluronic acid membrane (*n* = 25) or collagen powder (*n* = 23) before implantation in the defect in a single step procedure. 48 patients were examined clinically and radiologically after an average of 29 months postoperatively. The clinical scores revealed a significant improvement compared to postoperative scores whereas in the MRI and histology of second-look arthroscopies none showed complete hyaline cartilage. In the 20 patients with MSC therapy of the femur condyle satisfactory clinical results (IKDC 90.4 points) were also reported after an average of 29 months postoperatively. The MRI showed a satisfactory integration of the graft in 80% of the patients. Instead of direct defect coverage, some groups describe a simple intra-articular injection of MSC [113], with the intention of the ability of homing of the MSC. Centeno et al. report about an injection in a patient with early osteoarthritis of the knee. In the MRI followup after 6 months, they revealed an increased cartilage volume compared to point of time before injection.

In summary, all applications for clinical use are based on very small case series. The MSC application technique was adopted from the clinical experience of autologous chondrocyte transplantation (fibrin, collagen gel, periosteal flap). Before a clinical use can be recommended, basic research to optimize application techniques, cell preparation, and concentration are essential [114]. With improved knowledge from basic studies further evaluation of the clinical potential of MSC application has to be performed in larger randomized controlled trials.

### 5.3. Bone

In bone, the main focus of regenerative medicine approaches lies on atrophic non union and replacement of lost bone tissue. Large bone defects are usually caused by trauma, infection, or tumors, as atrophic nonunion are usually caused by insufficient blood supply, interposition of soft tissue or consequence after infection. Current treatment strategies include autologous bone grafts from the iliac crest, which is actually the gold standard—and as salvage procedures—autologous fibula graft transfer and allogenic bone graft transplantation. However, all mentioned techniques show limitations, as bone supply is limited, autologous bone harvesting is accompanied with high rates of morbidity and allogenic transplantation inheres the risk of transmission of diseases or rejection [115, 116].

In the last two decades, regenerative medicine approaches have been extensively studied to improve bone healing, or even generate functional bone tissue to substitute lost bone. Many *in vitro* studies were performed to investigate applicability of different stem cell types for bone regeneration. Here, promising capacity for differentiating towards bone cells, enhancing bone healing and vascularization could be proven for embryonic stem cells and different adult mesenchymal stem cells. However, due to the ethical and safety concerns mentioned above, only adult stem cells are presently taken into consideration for therapeutic applications [63]. Here, mesenchymal stem cells presently seem to be the most promising candidates for bone regeneration, due to their excellent osteogenic differentiation capacity [69].

*In vitro* trials found out that MSC strongly promote angiogenesis by paracrine factors after mechanical stimulation, as occurring during fracture healing [117], which makes MSC more interesting for bone regeneration. This paracrine enhancement of angiogenesis in bone regeneration could also be confirmed in animal models *in vivo* [118].

The capacity of mesenchymal stem cells for homing to injured tissues known from other fields was also demonstrated for fractures. Here, mesenchymal stem cells showed migration towards the fracture site after systemic application in a mouse model. The study further revealed that the cells enriched there and participated in fracture healing by paracrine induction of tissue healing, reduction of systemic and local inflammation and differentiating into bone cells [74]. However, the majority of the stem cells were trapped in the lungs after systemic application, thus making local application more practicable for bone regeneration [119].

Different groups achieved to compose small bone-like tissue constructs *in vitro*, by composing MSC with a variety of different biomaterials. Implanted into animals, several of these constructs survived *in vivo* [120]. However, researchers did not succeed in composing vital bone pieces in larger volumes, or even whole bones. This is due to the diffusion tract being larger than 200 *μ*m. Beyond 200 *μ*m, diffusion is not sufficient for providing cells with oxygen and nutrients. Therefore, functional vascularization is a prerequisite for survival of such solid tissues. Up to now, the problem of vascularization in tissue engineering is not yet solved, inhibiting the translation of tissue engineering methods into the clinic [43].

Nonetheless, regenerative medicine for bone healing has reached the patient in form of cell therapy approaches to treat localized bone defects or systemic diseases of the skeleton [39]. Here, autologous bone marrow or autologous mesenchymal stem cells was successfully implanted in a number of patients to enhance fracture/osteotomy healing, fill bone defects, treat pseudarthrosis, bone cysts, osteonecrosis, or enhance spinal fusion. Relevant clinical applications are summarized in Table 1.

## 6. Conclusions

Current data provides a number of interesting approaches to treat musculoskeletal pathologies with the support of mesenchymal stem cells. But considering the limited, partially only preclinical data we believe that a standardized clinical application will take at least an additional 5 to 10 years. In order to realize the full therapeutic potential of stem cells, a number of open questions has to be to be answered. Besides the necessity of establishing further data about native stem cell function and pathways, basic research in the understanding of native tendon, bone, and cartilage regeneration also has to be continued. Especially signal pathways have to be understood because single-MSC application might be insufficient or only partially sufficient without the adequate signal for inducing tissue regeneration. The regenerated tissue also has to provide the appropriate 3-dimensional structure including production of extracellular matrix and biomechanical behavior according to native tissue. Therefore, tissue engineering will play an important role in the next years. In the near future, an interdisciplinary approach with biologists, bioengineers, and clinicians will be essential to achieve the clinical application of mesenchymal stem cells.

## Figures and Tables

**Table 1:** Clinical applications of mesenchymal stem cells in bone regeneration.

Author	Diagnosis	Application	*n* patients	Results
Treatment of nonunions				

Connolly et al. 1991 [49]	Atrophic pseudarthrosis	Percutaneous autologous bone marrow injection	20	Healing capacity comparable to autologous cancellous bone grafting

Garg et al. 1993 [50]	Nonunion in long bones	Percutaneous autologous bone marrow injection	20	17 out of 20 cases united in 5 months

Kettunen et al. 2002 [51]	Tibially delayed or non-union	Percutaneous autologous bone marrow injection	41	Appeared to be as effective as open techniques

Hernigou et al. 2005 [52]	Atrophic pseudarthrosis	Percutaneous autologous bone marrow injection	60	Application is effective and safe Positive correlation between number of progenitor cells and callus volume

Goel et al. 2005 [53]	Tibial non-union	Percutaneous autologous bone marrow injection	20	15 out of 20 patients showed bone union

Treatment of osteonecrosis				

Hernigou and Beaujean 2002 [54]	Osteonecrosis femoral head	Injection of autologous bone marrow concentrate	116 (189 hips)	Very good results in early stages Injection of greater number of progenitor cells transplanted had better outcomes

Gangji et al. 2004 [55]	Osteonecrosis femoral head	Injection of autologous bone marrow concentrate	13 (18 hips)	Significant reduction of pain, progression and improvement of function

Hernigou et al. 2009 [56]	Osteonecrosis femoral head	Injection of autologous bone marrow concentrate	342 (534 hips)	High amount of progenitor cells as predictor for successful outcome

Enhancing spinal fusions				

Neen et al. 2006 [57]	Spinal fusions	Autologous bone marrow aspirate on hydroxyapatite-collagen I-composite	50	Similar healing capacity as autologous cancellous bone grafting in posterolateral fusion Inferior results in interbody fusions

Gan et al. 2008 [58]	Spinal fusions	Bone marrow concentrate on tricalciumphosphate	41	After 34.5 months 95.1% cases showed good spinal fusion

Filling bone cysts				

Wright et al. 2008 [59]	Simple bone cysts	Intralesional injection of autologous bone marrow aspirate	77	Inferior results compared to injection of methylprednisolone

Park et al. 2008 [60]	Simple bone cysts	Implantation of autologous bone marrow aspirate implanted in combination with either nonvital allogenic bone graft or injected with bone powder	20 (23 cysts)	Injection of bone marrow-bone powder mix is effective alternative to open treatment

Zamzam et al. 2009 [61]	Simple bone cysts	Percutaneous autologous bone marrow injection	28	Application is a safe and effective treatment

Filling of bone defects				

Salama and Weissman 1978 [62]	Different bone defects	Xenograft with bone marrow aspirate	28	Results have been “most satisfactory”

Jäger et al. 2009 [63]	volumetric bone deficiencies	local autologous bone marrow/mesenchymal stem cell injection	10	May be a promising alternative to autogenous bone grafting

Marcacci et al. 2007 [64]	Large bone diaphysis defect	autologous MSCs were expanded *in vitro* and seeded on hydroxyapatite scaffolds	4	Followup up to 7 years after surgery, good integration of implant, no secondary fractures

Various applications				

Hendrich et al. 2009 [65]	various bone healing disturbances	Bone marrow concentrate	101	Autogenous bone marrow concentrate application is safe

Giannini et al. 2009 [66]	Osteochondral talus defects	arthroscopic-assisted injection of autologous bone marrow aspirate	48	Functional improvement

Dallari et al. 2007 [67]	High tibial osteotomy	Lyophilized bone chips with platelets-enriched plasma with bone marrow aspirate	33	Lyophilized bone chips with platelets-enriched plasma with or without bone marrow aspirate enhance healing

Kitoh et al. 2009 [68]	femoral and tibial lengthenings	Application of MSC expanded *in vitro* with PRP	28 (51 osteotomies)	No enhancement of bone healing by MSC/PRP
